# Astaxanthin Ameliorated Parvalbumin-Positive Neuron Deficits and Alzheimer’s Disease-Related Pathological Progression in the Hippocampus of *App^NL-G-F/NL-G-F^* Mice

**DOI:** 10.3389/fphar.2020.00307

**Published:** 2020-03-11

**Authors:** Nobuko Hongo, Yusaku Takamura, Hiroshi Nishimaru, Jumpei Matsumoto, Kazuyuki Tobe, Takashi Saito, Takaomi C. Saido, Hisao Nishijo

**Affiliations:** ^1^System Emotional Science, Faculty of Medicine, University of Toyama, Toyama, Japan; ^2^First Department of Internal Medicine, Faculty of Medicine, University of Toyama, Toyama, Japan; ^3^Laboratory for Proteolytic Neuroscience, RIKEN Center for Brain Science, Wako-shi, Japan; ^4^Department of Neurocognitive Science, Institute of Brain Science, Nagoya City University Graduate School of Medical Science, Nagoya, Japan

**Keywords:** astaxanthin, Alzheimer’s disease, amyloid β, parvalbumin-positive neuron, hippocampus, hyperphosphorylated tau, glutathione, 4-HNE protein

## Abstract

Growing evidence suggests that oxidative stress due to amyloid β (Aβ) accumulation is involved in Alzheimer’s disease (AD) through the formation of amyloid plaque, which leads to hyperphosphorylation of tau, microglial activation, and cognitive deficits. The dysfunction or phenotypic loss of parvalbumin (PV)-positive neurons has been implicated in cognitive deficits. Astaxanthin is one of carotenoids and known as a highly potent antioxidant. We hypothesized that astaxanthin’s antioxidant effects may prevent the onset of cognitive deficits in AD by preventing AD pathological processes associated with oxidative stress. In the present study, we investigated the effects of astaxanthin intake on the cognitive and pathological progression of AD in a mouse model of AD. The *App^NL-G-F/NL-G-F^* mice were fed with or without astaxanthin from 5-to-6 weeks old, and cognitive functions were evaluated using a Barnes maze test at 6 months old. PV-positive neurons were investigated in the hippocampus. Aβ42 deposits, accumulation of microglia, and phosphorylated tau (pTau) were immunohistochemically analyzed in the hippocampus. The hippocampal anti-oxidant status was also investigated. The Barnes maze test indicated that astaxanthin significantly ameliorated memory deficits. Astaxanthin reduced Aβ42 deposition and pTau-positive areal fraction, while it increased PV-positive neuron density and microglial accumulation per unit fraction of Aβ42 deposition in the hippocampus. Furthermore, astaxanthin increased total glutathione (GSH) levels, although 4-hydroxy-2,3-trans-nonenal (4-HNE) protein adduct levels (oxidative stress marker) remained high in the astaxanthin supplemented mice. The results indicated that astaxanthin ameliorated memory deficits and significantly reversed AD pathological processes (Aβ42 deposition, pTau formation, GSH decrease, and PV-positive neuronal deficits). The elevated GSH levels and resultant recovery of PV-positive neuron density, as well as microglial activation, may prevent these pathological processes.

## Introduction

Alzheimer’s disease (AD) is the prevailing form of dementia, in which memory loss is the first symptom reported by patients (Jahn, 2013). The histopathologic features of the brain with AD are senile plaques that are composed of aggregated β-amyloid peptides (Aβ) and associated proteins and neurofibrillary tangles that are composed of phosphorylated tau (pTau) (Selkoe and Hardy, 2016). There are two major forms of Aβ: Aβ40 and Aβ42. Aβ42 is more neurotoxic due to its higher hydrophobicity, which promotes oligomerization and aggregation (Blennow and Zetterberg, 2018). Aβ deposition also induces microglial activation, which may ameliorate neurodegeneration due to Aβ accumulation (Deczkowska et al., 2018; Edwards, 2019). Accumulating evidence suggests that oxidative stress is implicated in AD; Aβ generates reactive oxygen species leading to mitochondrial dysfunctions *in vitro* (Lustbader et al., 2004; Manczak et al., 2010). A human study on mild cognitive impairment and AD reported that reduction of glutathione (GSH) with anti-oxidative action was observed in the hippocampus and frontal cortex, which was correlated with cognitive deficits (Mandal et al., 2015), while 4-hydroxy-2,3-trans-nonenal (4-HNE) protein adduct levels (a marker of lipid peroxidation) were elevated in AD patients (Markesbery and Lovell, 1998; Zarkovic, 2003).

A subclass of GABAergic interneurons co-expresses the calcium-binding protein parvalbumin (PV). Fast-spiking PV-positive neurons facilitate sensory and cognitive information processing by controlling pyramidal neuron activity and generating gamma oscillation (Bartos et al., 2007; Sohal et al., 2009; Nguyen et al., 2011; Nakamura et al., 2015). PV-positive neurons are sensitive to oxidative stress (Jiang et al., 2013; Kann et al., 2014; Steullet et al., 2017), and number of PV-positive neurons was reduced in the hippocampus of AD mouse models as well as AD patients (Takahashi et al., 2010). Furthermore, reduction of gamma oscillation associated with its dysfunction or phenotype loss was reported in human AD patients (Stam et al., 2002) and human amyloid precursor protein (hAPP) transgenic mice (Verret et al., 2012), which may be implicated in cognitive deficits in the hAPP mice and possibly in AD patients (Verret et al., 2012).

Astaxanthin is one of the carotenoids, naturally distributed in crustanceans, such as shrimps and crabs, and fish such as salmons and sea bream (Miki et al., 1982; Matsuno, 2001), and known as a highly potent antioxidant (Miki, 1991; Rodrigues et al., 2012). Recent clinical studies reported that astaxanthin may improve cognitive functions in aged individuals (Katagiri et al., 2012) and that astaxanthin supplementation decreased Aβ and phospholipid peroxides in red blood cells in healthy senior subjects (Nakagawa et al., 2011; Kiko et al., 2012). The previous available data suggest that astaxanthin may have a therapeutic or preventive effect on the progression of AD. Therefore, we hypothesized that astaxanthin’s anti-oxidant effects may contribute to the prevention of the onset of cognitive deficits in AD through its effects on Aβ accumulation, pTau, microglia, and PV-positive neurons. In the present study, the effects of astaxanthin intake on cognitive functions, histopathological progression of AD, and PV-positive neurons were investigated in a mouse model of AD with single App knock-in, which is free from side effects due to overexpression of amyloid precursor protein (APP) (Saito et al., 2014; Sasaguri et al., 2017; Hashimoto et al., 2019)

## Materials and Methods

### Experimental Schedule

Our previous study reported that cortical Aβ deposition in *App*^NL-G-F/NL-G-F^ mice (*App^NL-G-F^* mice) used in this study began by 2 months old and the *App^NL-G-F^* mice developed cognitive impairment at 6 months old, while microgliosis was observed at 9 months old (Saito et al., 2014). In order to evaluate preventive effects of astaxanthin on AD-related pathological progression, administration of astaxanthin to *App^NL-G-F^* mice started before formation of Aβ deposition, and the mice were tested with a behavioral test for spatial memory (Barnes maze test) at 6 months old (see below for the details). To analyze effects of astaxanthin on histochemical and biochemical findings in the brain including microgliosis, the mice were sacrificed at 9 months old (see below for the details). Thus, feeding of astaxanthin-containing diet started after weaning at 5-to-6 weeks, and continued until sacrifice at 9 months old (see below for the details), while the mice were subjected to the Barnes maze test at 6 months old.

### Animals and Diets

The original lines of *App^NL-G-F^* mice were obtained from the RIKEN Center for Brain Science (Wako, Japan) and back-crossed onto a C57BL/6J background. After weaning at 5-to-6 weeks, male *App^NL-G-F^* mice were divided into two diet groups and fed normal chow (MF, Oriental Yeast Co. Ltd., Tokyo, Japan) with or without 0.02% astaxanthin as free form (w/w), which was derived from *Haematococcus pluvialis* (Fuji chemical industries Co., Ltd, Toyama, Japan). Age-matched male wild type (WT) C57BL/6J mice (Japan SCL Inc., Hamamatsu, Japan) were fed normal chow without astaxanthin. Thus, three groups of the mice were used in this study: (1) WT mice fed with normal chow without astaxanthin (control-fed WT mice, n = 25), (2) *App^NL-G-F^* mice fed with normal chow without astaxanthin (control-fed *App^NL-G-F^* mice, n = 23), and (3) *App^NL-G-F^* mice fed with normal chow with astaxanthin (astaxanthin-fed *App^NL-G-F^* mice, n = 24).

**Figure 1:**
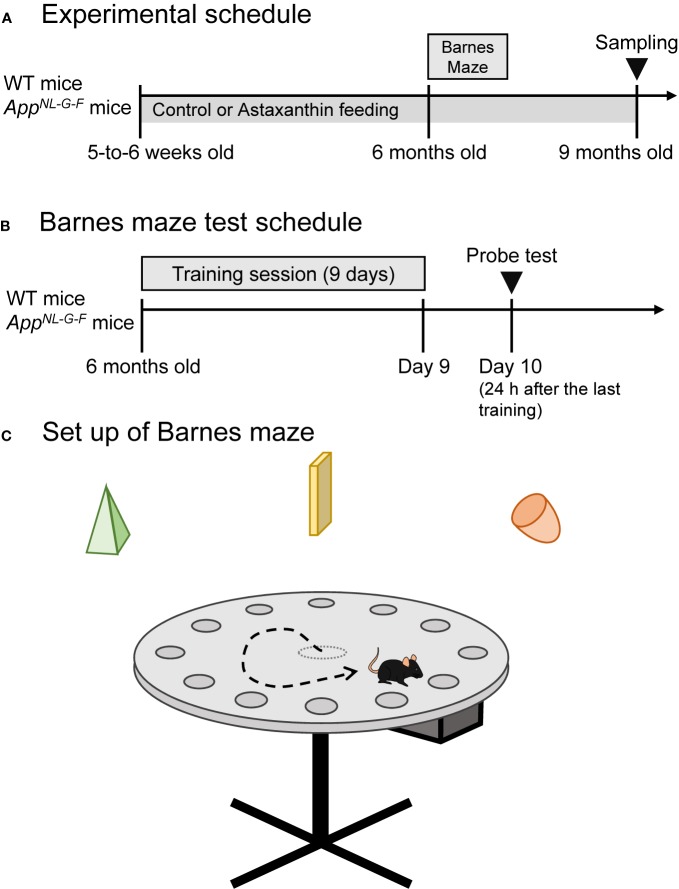
Experimental schedule and Barnes maze test. **(A)** Experimental schedule. **(B)** Barnes maze test schedule. **(C)** Set up of the Barnes maze test. Three extramaze (distant) cues were placed over the maze.

The *App^NL-G-F^* and WT mice were socially housed with mice on the same diet and the same genotype group in a constant temperature environment (22 ± 1°C) with a 12/12-h light/dark cycle (lights were turned on from 07:00 to 19:00). Food and water were available *ad libitum*. All mice were tested with a Barnes maze test at 6 months old and the same mice were sacrificed for immunohistochemical analyses and biochemical assays at 9 months old (see below) (**Figure 1A**). All experimental procedures were conducted according to the guidelines for care and use of laboratory animals approved by the University of Toyama and the National Institutes of Health’s Guide for the Care and Use of Laboratory Animals. This study was approved by the Ethics Committee for Animal Experiments at the University of Toyama (Permit No. A2013MED-53).

### Barnes Maze Test

A total of 72 mice were tested with the Barnes maze test (control-fed WT mice, n = 25; control-fed *App^NL-G-F^* mice, n = 23; astaxanthin-fed *App^NL-G-F^* mice, n = 24). In an initial training session, two trials per day were performed continuously for 9 days (**Figure 1B**). In the training session, each mouse was placed in the center of a grey circular table (diam. = 1.0 m), which had 12 holes around the perimeter (**Figure 1C**). The circular open table was 75 cm above the floor and illuminated with 1,080 W lights. The mouse could escape into a black escape box (17 × 13 × 7 cm) with paper bedding, which was located under one of the holes. The location of the hole with the escape box (goal) was the same in a given mouse but randomly different across individual mice. After each trial, the maze surface and escape box were cleaned with 70% ethanol. The maze was rotated daily, with the spatial location of the goal hole consistent in reference to the extra-maze room cues to prevent a bias according to intra-maze local cues. Escape latencies to the goal hole in the training session were measured by Time BCM (O'hara & Co., Tokyo, Japan).

One day after the last training day, each mouse was tested with a probe test (PT) (**Figure 1B**). Each mouse was placed on the table without the escape box for 3 min. In the PT, two parameters were evaluated. The goal hole region was defined as a belt-shaped area with a width of 4.7 cm around the goal hole (diam. = 5.0 cm). The number of visits to the goal hole region was defined as the number of times the center of gravity of a given mouse image crossed the goal hole region during 3 min of the PT. The goal hole time (sec) was defined as the time during which the center of gravity of a given mouse image stayed in the goal hole region. Data of the mice that fell off the table were excluded from the analysis.

### Sampling and Preparation of Brain Specimens

Brain specimens were prepared from the mice used for the Barnes maze test. Under deep anesthesia with a mixture of three different anesthetics (medetomidine, midazolam, and butorphanol; 0.75, 4.0, and 5.0 mg/kg body weight, respectively; i.p.), the mice were transcardially perfused with heparinized saline (0.9% NaCl). After perfusion, the brain was removed from the skull. The 47 right hemispheres were used for the measurements of biochemical markers (4-HNE, GSH, and Aβ42). The hippocampus and prefrontal cortex (PFC) corresponding to the prelimbic and infralimbic areas were dissected from the right hemisphere and stored −80°C. The hippocampus and PFC were sonicated in 50 mM Tris-HCl buffer, pH 7.6, 150 mM NaCl, and the protease inhibitor cocktail (complete protease cocktail, Merck KGaA, Darmstadt, Germany) and centrifuged at 200,000 × g for 20 min at 4°C. The supernatant was collected as soluble fraction. The remaining pellet was sonicated in 50 mM Tris-HCl buffer, pH 7.6, containing 6 M guanidine–HCl and 150 mM NaCl, and centrifuged at 200,000 × g for 20 min at 4°C. The supernatant was collected as insoluble fraction. The protein content of each fraction was determined by BCA assay kit (Thermo Fisher Scientific Inc., MA, USA). Soluble and/or insoluble fractions were used for measurements of Aβ42, 4-HNE protein adduct, and total GSH (see below).

The 42 left hemispheres were used for PV immuno-histochemistry, while 15 right and left hemispheres were used for immunohistochemistry of Aβ42, pTau, and Iba1 in the brain sections. These hemispheres were fixed in 4% paraformaldehyde dissolved in 0.1 M phosphate buffer (PB; pH 7.4) overnight, and used for immunohistochemistry (see below).

### Quantitative Measurement of Aβ Deposition (ELISA)

A total of 36 mice were used (control-fed WT mice, n = 14; control-fed *App^NL-G-F^* mice, n = 9; astaxanthin-fed *App^NL-G-F^* mice, n = 13). The insoluble fraction samples were used to measure the amounts of Aβ42 by sandwich ELISA (Human β Amyloid (1–42) ELISA Kit Wako, FUJIFILM Wako Pure Chemical Corporation, Osaka, Japan).

### Quantification of 4-HNE Protein Adduct (Slot Blot)

A total of 32 mice were used (control-fed WT mice, n = 12; control-fed *App^NL-G-F^* mice, n = 10; astaxanthin-fed *App^NL-G-F^* mice, n = 10). The soluble and insoluble fraction samples were loaded on the PVDF membrane (Immobilon P, Merck KGaA, Darmstadt, Germany) using the slot blot manifold. For standard 4-HNE protein adduct, BSA at the concentration of 1 mg/ml was treated with 100 μmol/L 4-HNE at 37°C for 4 h. Four-HNE monoclonal antibody (clone HNEJ-2, JaICA, Shizuoka, Japan) was used as the first antibody. Peroxide labeled-anti mouse IgG antibody (SeraCare Life Sciences Inc., MA, USA) was used as the secondary antibody. The amount of 4-HNE protein adduct was quantified by a luminol reagent kit (ECL, GE Healthcare, Ill, USA). The luminescence was detected by cooled CCD imager (LAS400, GE Healthcare) and analyzed using ImageJ ver.1.8.0 (Rasband, W.S., ImageJ, NIH, Bethesda, USA, https://imagej.nih.gov/ij/, 1997–2018.).

### Quantitative Measurement of Total GSH

A total of 29 mice were used (control-fed WT mice, n = 10; control-fed *App^NL-G-F^* mice, n = 9; astaxanthin-fed *App^NL-G-F^* mice, n = 10). Sulfosalicylic acid (1% of final concentration) was added to the soluble fraction. The mixture was centrifuged at 8000 × g for 10 min at 4°C. The 1 N NaOH was added to the supernatant to 9% volume for deacidification. The sample was reacted with 25 μg/ml DTNB (5-5'-dithiobis[2-nitrobenzoic acid], Dojindo Laboratories, Kumamoto, Japan), 40 μg/ml NADPH, and 1 U/ml GSH reductase (Oriental Yeast Co. Ltd., Tokyo, Japan) for 10 min at 37°C. Then, total GSH was measured by colorimetric absorbance at 405 nm.

### Immunohistochemistry and Analysis of PV-Positive Neurons

A total of 42 mice were used (control-fed WT mice, n = 13; control-fed *App^NL-G-F^* mice, n = 14; astaxanthin-fed *App^NL-G-F^* mice, n = 15). PV-positive neurons were stained following the same protocol described in our previous studies (Nguyen et al., 2011; Urakawa et al., 2013; Nakamura et al., 2015; Jargalsaikhan et al., 2017). Briefly, the fixed blocks of the left hemispheres were cut into 40-μm-thick sections. Five serial sections were collected for every 200 μm; one was used for PV staining, and one was used for cresyl-violet staining. The sections were stained with mouse monoclonal anti-PV antibodies (1:10,000 dilution in 1% horse serum PBS, Sigma, St. Louis, MO, USA).

PV-positive neurons were analyzed according to our previous studies (Nakamura et al., 2015; Jargalsaikhan et al., 2017). Briefly, the brain sections were observed using an all-in-one fluorescence microscope system (BZ-9000, Keyence Corporation, Osaka, Japan). PV-positive neurons were counted in the five sections in the hippocampus at −1.60, −1.76, −1.92, −2.08, and −2.24 mm posteriorly from the bregma based on the mouse brain atlas (Hof et al., 2000). The PV-positive neurons were counted using a stereological technique with systematic random sampling (StereoInvestigator v.7.53.1, MicroBrightField, Williston, VT, USA) (Sterio, 1984; Nakamura et al., 2015; Jargalsaikhan et al., 2017). The grid size for the analysis was set at 1741.60 × 719.55-μm, while the size for the counting frames with inclusion and exclusion lines was set at 200 × 200-μm. The software highlighted only PV-positive cell bodies within the counting frame without contact with the exclusion lines. We counted PV-positive objects in the counting frame only if they came into focus within a predetermined 5-μm thick optical dissector that was positioned 2 μm below the surface of the mounted section using the Z-axis microcator. The PV-positive neuron density was computed in each mouse.

### Immunofluorescent Staining and Analysis of Aβ42 and Iba1

A total of 15 mice were used (control-fed WT mice, n = 5; control-fed *App^NL-G-F^* mice, n = 5; astaxanthin-fed *App^NL-G-F^* mice, n = 5). Fixed right hemispheres were embedded in paraffin. Immunofluorescent staining was performed by Biopathology Institute Co., Ltd. (Oita, Japan). Anti-β-amyloid (1–42) antibody (rabbit IgG, AB5078P, MILLIPORE) and anti-Iba1 antibody (Goat IgG, ab5076, Abcam) were used as the primary antibodies. Anti-rabbit Alexa-594 and anti-goat Alexa 488 were used as the secondary antibodies. Finally, the brain sections were mounted on glass slides using mount media with DAPI (SlowFade Gold Antifade Reagent With DAPI, Thermo Fisher Scientific Inc., MA, USA).

Microscopic images of Aβ42 and Iba1 in the hippocampal sections at −1.80, −2.12, and −2.44 mm posteriorly from the bregma were captured under an identical, experimenter-blinded condition, using a fluorescent microscope (BX52, Olympus Corporation, Tokyo, Japan). The images were analyzed using ImageJ. Area fractions of Aβ42 of all images were estimated with binary data with the same threshold level (17,926/65,536). Area fractions of all Iba1 images were estimated with binary data with the same threshold level (17,408/65,536), excluding fractions below 19.6 μm^2^ particles.

### Immunohistochemistry and Analysis of Phosphorylated Tau (pTau)

A total of 15 mice were used (control-fed WT mice, n = 5; control-fed *App^NL-G-F^* mice, n = 5; astaxanthin-fed *App^NL-G-F^* mice, n = 5). Fixed hemispheres were embedded in paraffin. Immunohistochemical staining was performed by Biopathology Institute Co., Ltd. (Oita, Japan). MAPT/Tau (Ser198/Ser199/Ser202/Thr205) antibody (LS-C48043-50, Life Span Bioscience, Inc.) was used as the primary antibody. Then, the sections were treated with a polymer constituted with Fab’ fragment of anti-rabbit IgG antibody and peroxidase (Nichirei-histfine simple stain Max PO, Nichirei bioscience Inc., Tokyo, Japan). pTau was visualized with 3, 3'-diaminobenzidine and hydrogen peroxide. Finally, the sections were stained with hematoxylin-eosin.

Microscopic images of pTau in the hippocampal sections at −1.84, −2.16, and −2.48 mm posteriorly from the bregma were analyzed using ImageJ to estimate the fraction of pTau-positive areas. Stained regions of pTau were isolated by colorimetric intensity adjustment and then binarized with the threshold level (37,266/65,536), excluding fractions below 19.6 μm^2^ particles. Image processing and analyzing parameters were identical across the sections.

### Statistical Data Analysis

Data in the training session of the Barns maze test were compared among the three groups using repeated measures two-way ANOVA, followed by the Tukey *post hoc* test. The other data were compared among the three groups using one-way ANOVA, followed by the Tukey *post hoc* test. Additionally, pTau data were compared using pairwise t-test with Bonferroni adjustment (**Figure 8D**). The relationships between the two parameters were analyzed using simple regression analysis (**Figures 9A–C**, correlations between histological parameters for Iba1, pTau, and Aβ42; **Figures 9D–F**, correlations between the behavioral parameter in the PT of the Barnes maze test and histological parameters). *P* < 0.05 was considered statistically significant. The statistical analyses were performed using R ver.3.4.3 (R Core Team, 2017).

## Results

### Barnes Maze Test

**Figure 2:**
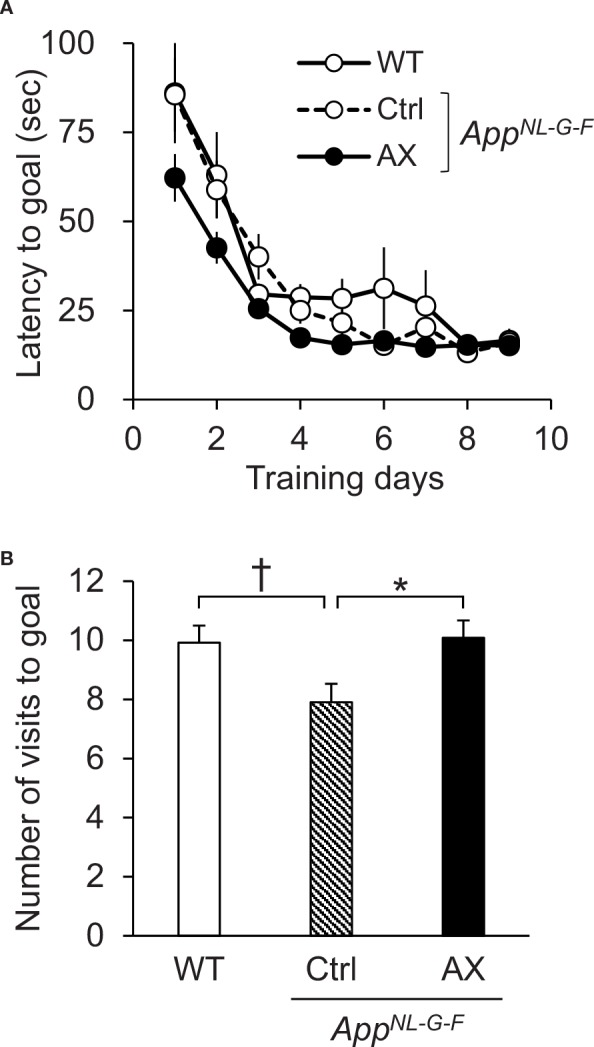
Performance in the Barnes maze test. **(A)** Average latency to escape to the goal hole in the training session. **(B)** Comparison of the number of visits to the goal region in the probe test (PT). WT, wild type C57BL/6J mice; *App^NL-G-F^*, *App^NL-G-F/NL-G-F^* mice; Ctrl, control-fed group; AX, astaxanthin-fed group. *P < 0.05; ^†^*P* < 0.1.

The Barnes maze test was used to assess the memory functions of the three groups of mice at 6 months old (**Figure 2**). In the initial training session, the average latency to escape to the goal hole gradually decreased across the 9 days in the three groups of the mice (**Figure 2A**). The statistical analysis by repeated measures two-way ANOVA (group × day) indicated that there was no significant main effect of group [F(2, 69) = 2.231, *P* = 0.115], nor interaction between group and day [F(16, 552) = 1.175, *P* = 0.284].

To assess spatial reference memory after the training, the escape box was removed in the PT 24 h after the last training day (**Figure 2B**). A comparison of the number of visits to the goal region by one-way ANOVA indicated that there was a significant difference in the number of visits to the goal region among the three groups [F(2, 67) = 3.912, *P* = 0.0247]. Post hoc multiple comparisons indicated that the number of visits to the goal region was significantly smaller in the control-fed *App^NL-G-F^* mice than the astaxanthin-fed *App^NL-G-F^* mice (Tukey test, *P* = 0.03603) and tended to be smaller than the control-fed WT mice (Tukey test, *P* = 0.05397). On the other hand, average goal hole time in the PT of the control-fed WT, control-fed *App^NL-G-F^*, and astaxanthin-fed *App^NL-G-F^* mice were 32.96 ± 2.35 (mean ± SEM), 26.05 ± 2.50, and 27.44 ± 2.28 sec, respectively. There was no significant difference among the three groups [F(2, 67) = 2.4152, *P* = 0.09709].

### PV-Positive Neuron Density

**Figure 3:**
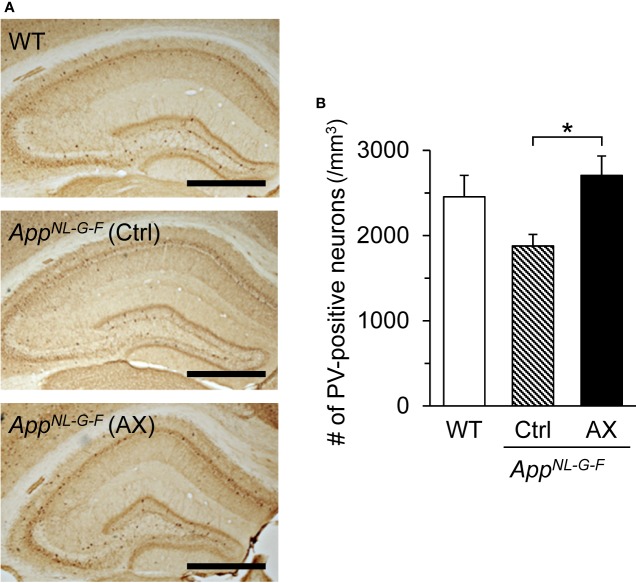
Comparison of the parvalbumin (PV)-positive neuron density in the hippocampus among the three groups of the mice. **(A)** Microscopic images of the hippocampus. The typical PV-immunohistochemical sections of the hippocampus in the control-fed WT, control-fed *App^NL-G-F^* (Ctrl), and astaxanthin-fed *App^NL-G-F^* (AX) mice are shown. The bar indicates 1,000 μm. **(B)** Comparison of PV-positive neuron density in the hippocampus. WT, wild type C57BL/6J mice; *App^NL-G-F^*, *App^NL-G-F/NL-G-F^* mice; Ctrl, control-fed group; AX, astaxanthin-fed group. *P < 0.05.

**Figure 3A** shows examples of PV-stained hippocampal sections in the three groups of mice at 9 months old. The mean densities of PV-positive neurons in the three groups are shown in **Figure 3B**. The mean density of PV-positive neurons in the control-fed *App^NL-G-F^* mice was decreased to about 75% of those in the control-fed WT mice and 70% of those in the astaxanthin-fed *App^NL-G-F^* mice. A statistical analysis using one-way ANOVA indicated that there was a significant main effect of group [F(2, 39) = 4.1877, *P* = 0.0225]. Post hoc tests indicated that the mean cell density was significantly higher in the astaxanthin-fed *App^NL-G-F^* mice than in the control-fed *App^NL-G-F^* mice (Tukey test, *P* = 0.01922).

### Quantification of Aβ42 Levels by ELISA

**Figure 4:**
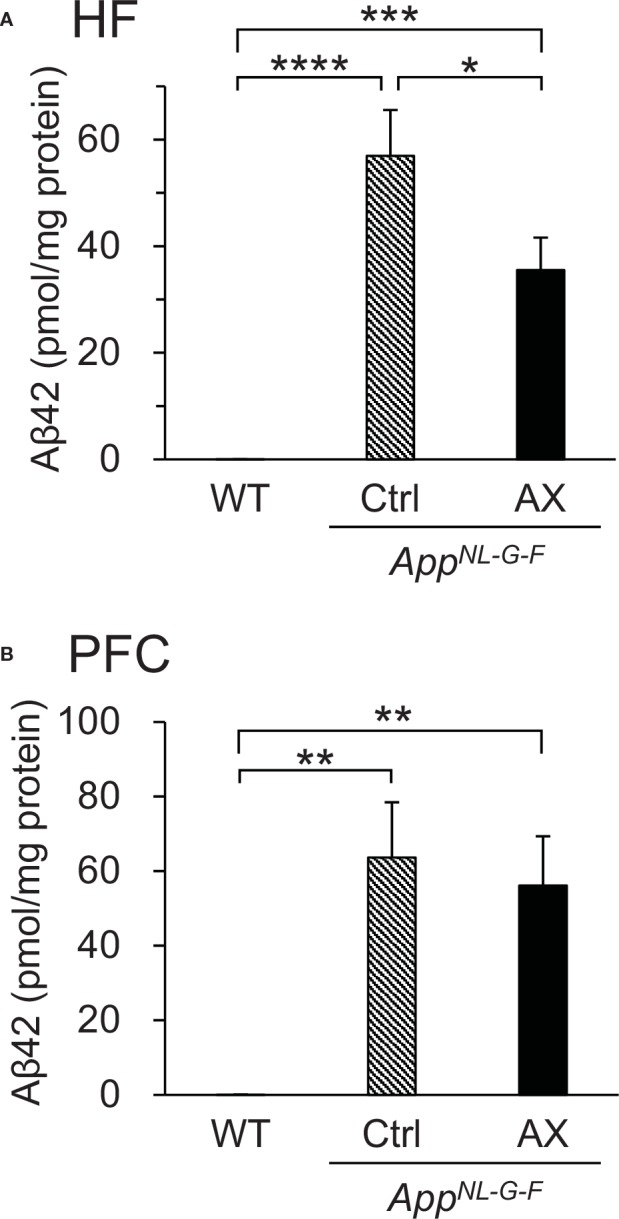
Comparison of Aβ42 levels quantified by ELISA among the three groups of mice. **(A)** Comparison of Aβ42 levels in the hippocampus (HF). **(B)** Comparison of Aβ42 levels in the prefrontal cortex (PFC). WT, wild type C57BL/6J mice; *App^NL-G-F^*, *App^NL-G-F/NL-G-F^* mice; Ctrl, control-fed group; AX, astaxanthin-fed group. *P < 0.05; **P < 0.01; ***P < 0.001; ****P < 0.0001.

The Aβ42 levels in the hippocampus and PFC were quantified in the *App^NL-G-F^* and WT mice at 9 months old by ELISA (**Figure 4**). In the hippocampus (**Figure 4A**), there was a significant difference in Aβ42 levels among the three groups [F(2.0, 12.8) = 36.811, *P* < 0.0001]. Post hoc multiple comparisons indicated that Aβ42 levels were significantly higher in the control-fed *App^NL-G-F^* mice (Tukey test, *P* < 0.0001) and astaxanthin-fed *App^NL-G-F^* mice (Tukey test, *P* < 0.0001) than in the control-fed WT mice. Furthermore, Aβ42 levels were significantly higher in the control-fed *App^NL-G-F^* mice than in the astaxanthin-fed *App^NL-G-F^* mice (Tukey test, *P* = 0.02925). In the PFC (**Figure 4B**), Aβ42 levels were also increased in the *App^NL-G-F^* mice. There was a significant difference in Aβ42 levels among the three groups [F(2.0, 12.8) = 17.298, *P* = 0.0002]. Post hoc multiple comparisons indicated that Aβ42 levels were significantly higher in the control-fed *App^NL-G-F^* mice (Tukey test, *P* = 0.00069) and astaxanthin-fed *App^NL-G-F^* mice (Tukey test, *P* = 0.00089) than in the control-fed WT mice.

### Oxidative and Anti-Oxidant Status in the Hippocampus

**Figure 5:**
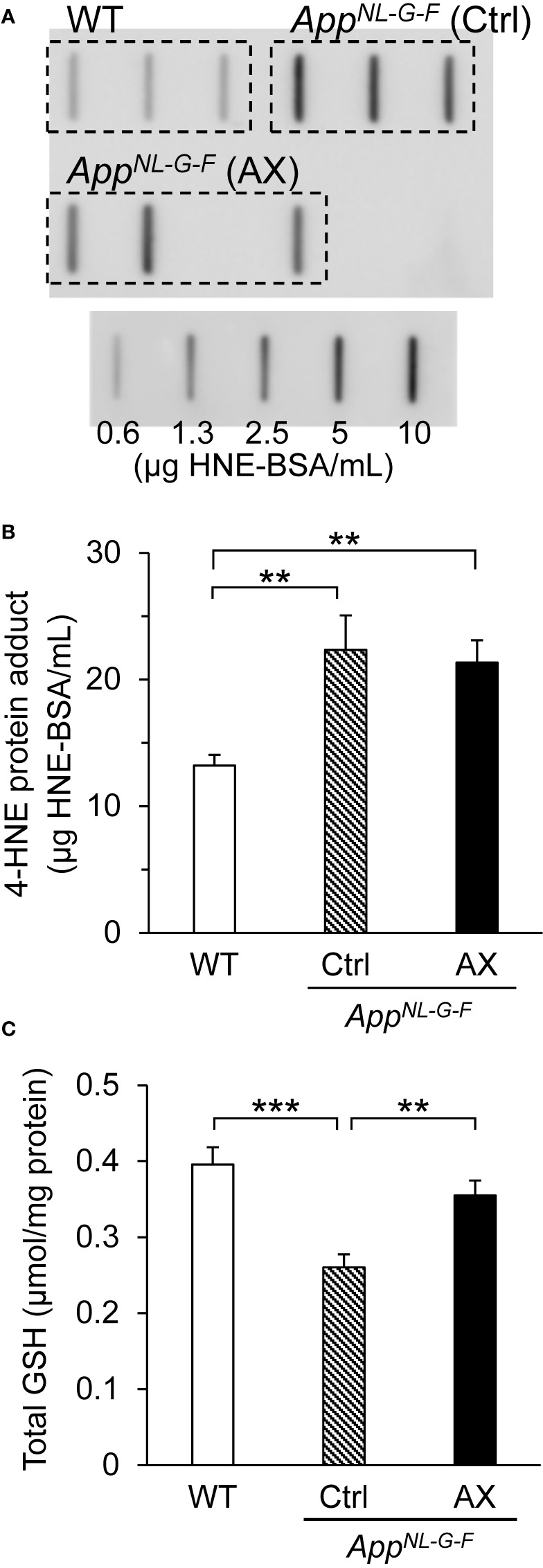
Comparison of oxidative and anti-oxidant status in the hippocampus among the three groups of mice. **(A)** Examples of slot bot images. **(B)** Comparison of 4-hydroxy-2,3-trans-nonenal (4-HNE) protein adduct levels (oxidative stress marker) quantified by slot blot analysis. **(C)** Comparison of total glutathione (GSH) levels measured by GSH reductase recycling and Ellman’s reagent. WT, wild type C57BL/6J mice; *App^NL-G-F^*, *App^NL-G-F/NL-G-F^* mice; Ctrl, control-fed group; AX, astaxanthin-fed group. **P < 0.01; ***P < 0.001.

Oxidative stress due to Aβ42 accumulation was assessed by quantifying 4-HNE bound to proteins (**Figures 5A, B**). Examples of slot blot analyses of 4-HNE protein adduct in three mice from each group are shown in **Figure 5A**. A statistical analysis (one-way ANOVA) indicated a significant difference among the three groups [F(2.0, 15.1)=11.722, *P* = 0.00084] (**Figure 5B**). Post hoc multiple comparisons indicated that 4-HNE protein adduct levels were significantly higher in the control-fed *App^NL-G-F^* mice (Tukey test, *P* = 0.00350) and astaxanthin-fed *App^NL-G-F^* mice (Tukey test, *P* = 0.00960) than in the control-fed WT mice.

To assess anti-oxidant status in the hippocampus, we compared total GSH levels in the hippocampus among the three groups of the mice (**Figure 5C**). A statistical analysis (one-way ANOVA) indicated a significant difference among the three groups [F(2, 26)=11.61, *P* = 0.00025]. Post hoc multiple comparisons indicated that the total GSH levels in the hippocampus were significantly lower in the control-fed *App^NL-G-F^* mice than that in the control-fed WT mice (Tukey test, *P* = 0.00020) and astaxanthin-fed *App^NL-G-F^* (Tukey test, *P* = 0.00767), and there was no significant difference in the total GSH levels between the control-fed WT and the astaxanthin-fed *App^NL-G-F^* mice (Tukey test, *P* = 0.32454).

### Aβ42 Deposition and Microglial Accumulation

**Figure 6:**
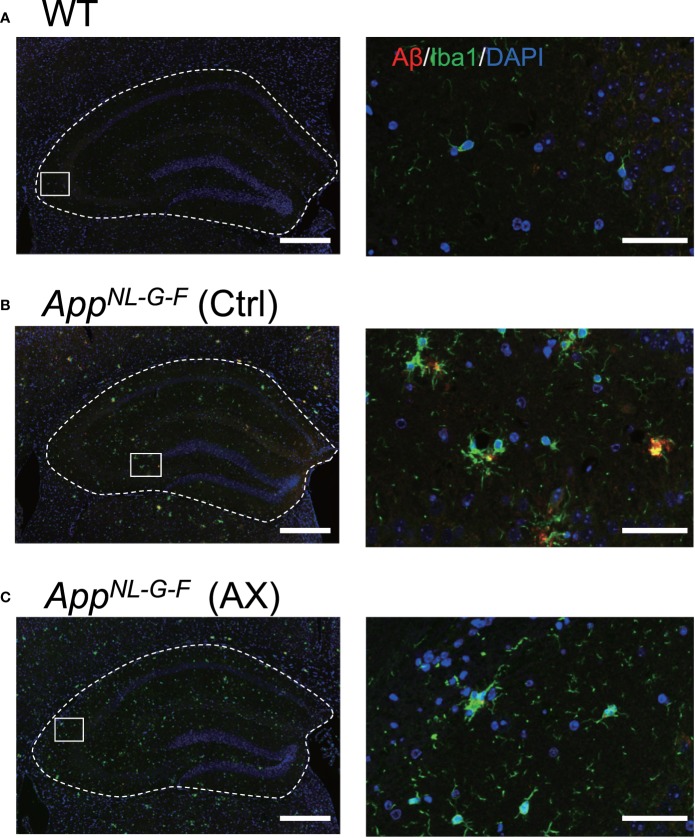
Co-localization of Aβ42 and Iba1 in *App^NL-G-F^* mice. Typical immunohistofluorescence images of Aβ42 and Iba1 in the hippocampus of the control-fed WT **(A)**, control-fed *App^NL-G-F^* (Ctrl) **(B)** and astaxanthin-fed *App^NL-G-F^* (AX) **(C)** mice are shown. Red, green, and blue colors in each image indicate Aβ42, Iba1, and DAPI, respectively. The images on the right indicate enlarged images of the inset in the left images. Bars in the left and right panels represent 1,000 and 50 μm, respectively.

**Figure 7:**
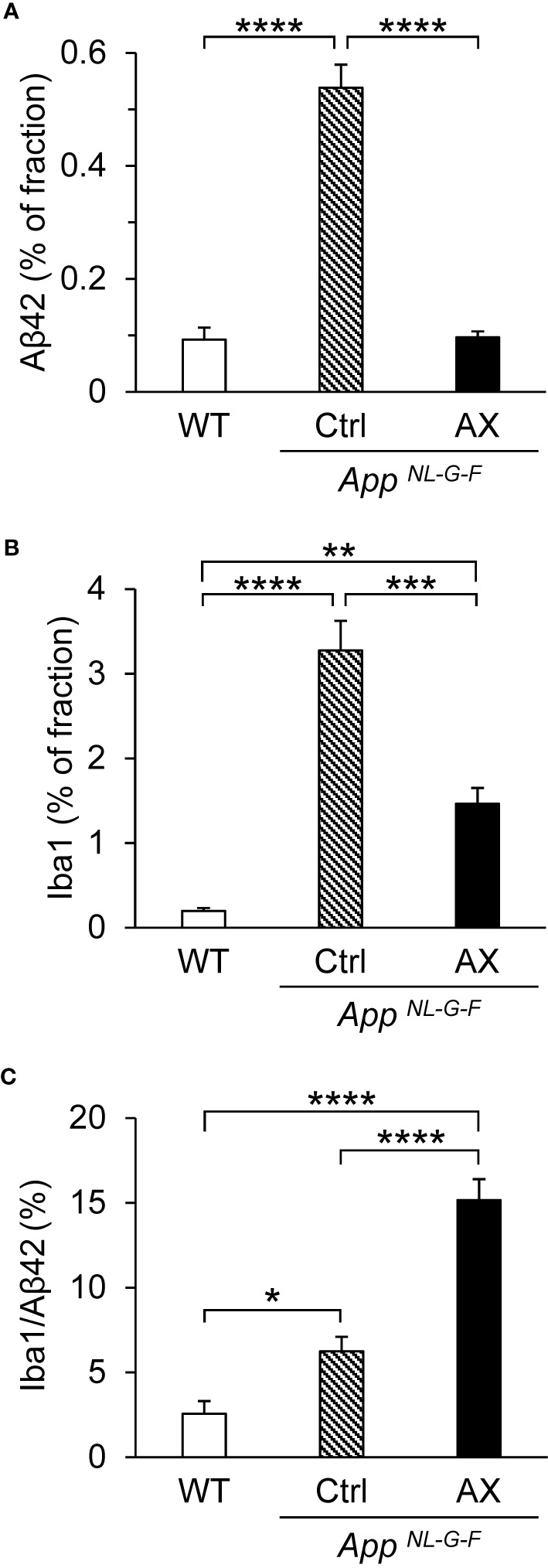
Comparison of the area fraction of Aβ42 **(A)**, area fraction of Iba1 **(B)**, and area fraction ratio of Iba1 against Aβ42 **(C)** in the hippocampus among the three groups of the mice. The hippocampal sections with immunohistochemical staining were analyzed by ImageJ. WT, wild type C57BL/6J mice; *App^NL-G-F^*, *App^NL-G-F/NL-G-F^* mice; Ctrl, control-fed group; AX, astaxanthin-fed group. *P < 0.05; **P < 0.01; ***P < 0.001; ****P < 0.0001.

We analyzed the relationships between Aβ42 deposition and microglial accumulation (**Figures 6** and **7**). Triple staining was performed using DAPI, antibody to Aβ42, and antibody to Iba1 (a marker of microgliosis) in the control-fed WT (**Figure 6A**), control-fed *App^NL-G-F^* (**Figure 6B**), and astaxanthin-fed *App^NL-G-F^* mice (**Figure 6C**). Aβ42 deposition (red) in the hippocampus colocalized with microglia (green) in the control-fed *App^NL-G-F^* mice. A statistical analysis of Aβ42 deposition by one-way ANOVA indicated a significant difference among the three groups [F(2, 12)=88.226, *P* < 0.0001] (**Figure 7A**). Post hoc multiple comparisons indicated that β42 deposition increased more in the control-fed *App^NL-G-F^* mice than in the control-fed WT mice (Tukey test, *P* < 0.0001) and astaxanthin-fed *App^NL-G-F^* mice (Tukey test, *P* < 0.0001). A statistical analysis of the microglial accumulation (Iba1 staining) by one-way ANOVA also indicated a significant difference among the three groups [F(2, 5.55)=52.963, *P* = 0.00024] (**Figure 7B**). Post hoc multiple comparisons indicated that Iba1 fraction was greater in the control-fed *App^NL-G-F^* mice (Tukey test, *P* < 0.0001) and astaxanthin-fed *App^NL-G-F^* mice (Tukey test, *P* = 0.00566) than in the control-fed WT mice. Furthermore, Iba1-fraction was smaller in the astaxanthin-fed *App^NL-G-F^* mice than in the control-fed *App^NL-G-F^* mice (Tukey test, *P* = 0.00033). Finally, we analyzed the colocalization of Aβ42 deposition and microglia. A statistical analysis of area fraction ratio of Iba1 against Aβ42 by one-way ANOVA also indicated a significant difference among the three groups [F(2, 12)=44.812, *P* < 0.0001] (**Figure 7C**). Post hoc multiple comparisons indicated that fraction ratios of Iba1 were greater in the control-fed *App^NL-G-F^* mice (Tukey test, *P* = 0.04796) and astaxanthin-fed *App^NL-G-F^* mice (Tukey test, *P* < 0.0001) than in the control-fed WT mice. Furthermore, fraction ratios of Iba1 were greater in the astaxanthin-fed *App^NL-G-F^* mice than in the control-fed *App^NL-G-F^* mice (Tukey test, *P* = 0.00008). These results indicated that microglia were more strongly accumulated in the astaxanthin-fed *App^NL-G-F^* mice than the control-fed *App^NL-G-F^* mice.

### Phosphorylated Tau Accumulation

**Figure 8:**
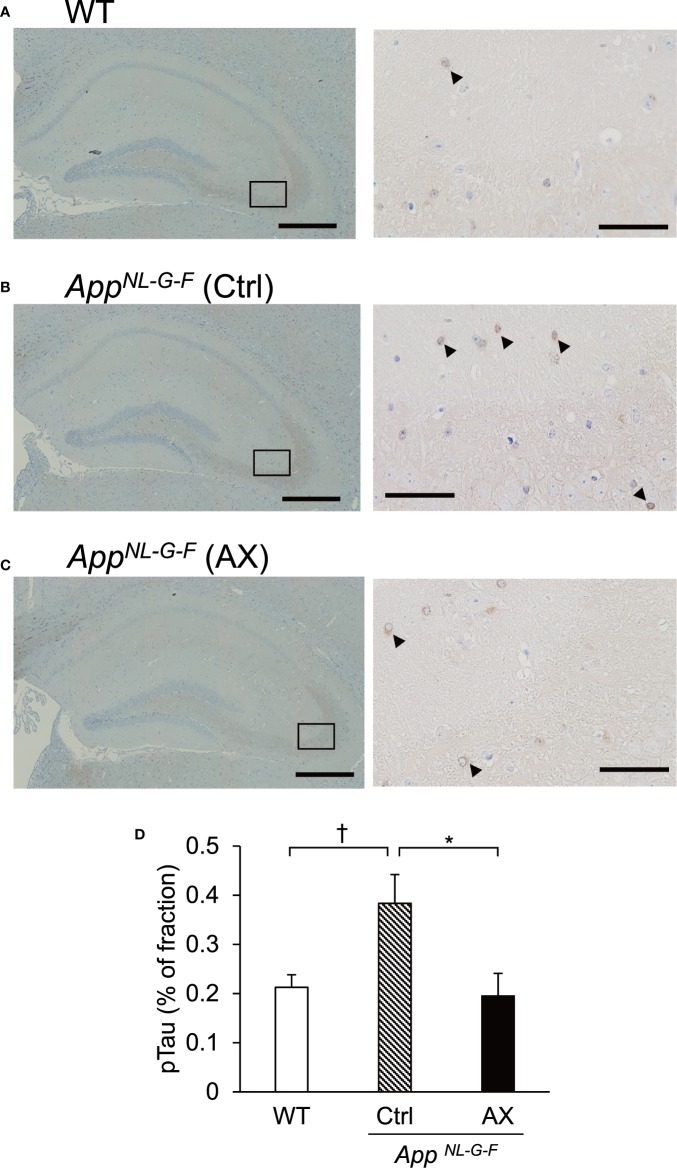
Comparison of area fraction of phosphorylated tau (pTau) in the hippocampus among the three groups of the mice. Typical images of pTau-positive cells in the hippocampus of the control-fed WT **(A)**, control-fed *App^NL-G-F^* (Ctrl) **(B)**, and astaxanthin-fed *App^NL-G-F^* (AX) **(C)** mice are shown. The images on the right indicate enlarged images of the inset in the left images. Bars in the left and right panels represent 1,000 and 50 μm, respectively. Arrowheads indicate pTau-positive cells. **(D)** Comparison of pTau fraction in the hippocampus among the three groups of the mice. The area fraction of pTau was analyzed using ImageJ. WT, wild type C57BL/6J mice; *App^NL-G-F^*, *App^NL-G-F/NL-G-F^* mice; Ctrl, control-fed group; AX, astaxanthin-fed group. ^†^*P* < 0.1; *P < 0.05.

The deposits of Aβ and the neurofibrillary tangles composed of hyperphosphorylated tau protein (pTau) are the neuropathological hallmarks of AD, and tauopathy is enhanced following Aβ amyloidosis (Hardy and Selkoe, 2002; Perrin et al., 2009; Hashimoto et al., 2019). Therefore, we immunohistochemically investigated the effects of astaxanthin on the tauopathy in the control-fed WT (**Figure 8A**), control-fed *App^NL-G-F^* (**Figure 8B**), and astaxanthin-fed *App^NL-G-F^* mice (**Figure 8C**). A statistical analysis of the pTau fraction using one-way ANOVA indicated that the difference among the three groups tended to be significant [F(2, 7.10)=3.6094, *P* = 0.08285]. Multiple comparisons by pairwise t-tests with Bonferroni correction indicated that pTau fraction tended to be higher in the control-fed *App^NL-G-F^* mice than the control-fed WT mice (*P* = 0.062) and that pTau fraction was significantly smaller in the astaxanthin-fed *App^NL-G-F^* mice than the control-fed *App^NL-G-F^* mice (*P* = 0.038) (**Figure 8D**).

### Correlation Analyses

**Figure 9:**
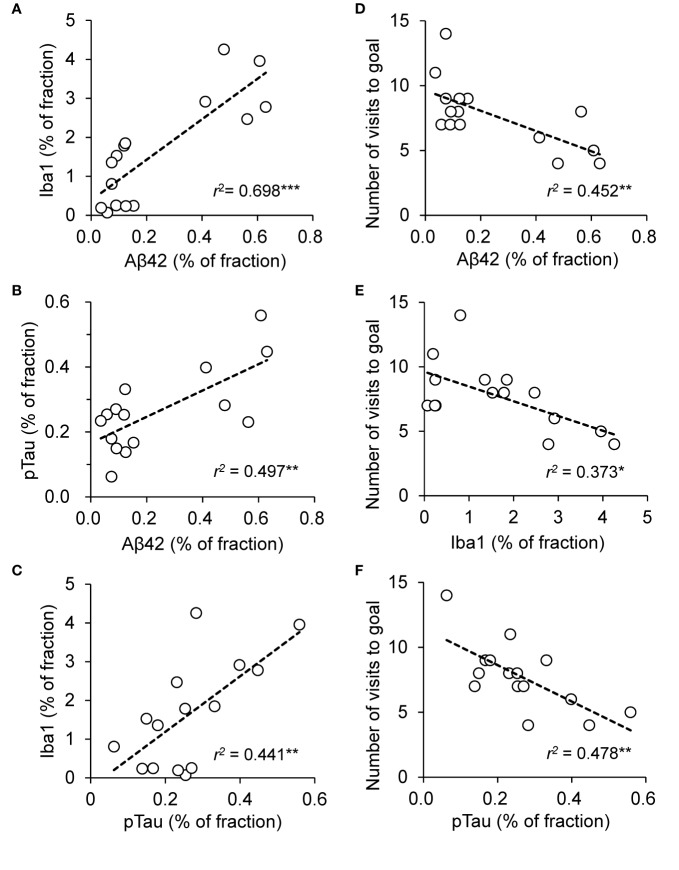
Relationships among Aβ42 fractions, Iba1 fractions, and phosphorylated tau (pTau) fractions in the hippocampus and performance in the probe test (PT) in the Barnes maze test across the three groups of the mice. **(A)** Significant positive correlation between Aβ42 and Iba1 fractions. **(B)** Significant positive correlation between Aβ42 and pTau fractions. **(C)** Significant positive correlation between pTau and Iba1 fractions. **(D)** Significant negative correlation between Aβ42 fraction in the hippocampus and number of visits to the goal region in the Barnes maze PT. **(E)** Significant negative correlation between Iba1 fraction in the hippocampus and number of visits to the goal region in the Barnes maze PT. **(F)** Significant negative correlation between pTau fraction in the hippocampus and number of visits to the goal region in the Barnes maze PT. “r” indicates Pearson’s product-moment correlation coefficient in the simple linear regression analysis. *P < 0.05; **P < 0.01 ; ***P = 0.001.

The above parameters in the AD pathology could be correlated each other according to the amyloid cascade theory. The area fraction of Aβ42 was significantly and positively correlated with Iba1 fraction [F(1, 13) = 30.0, *P* = 0.00011] (**Figure 9A**) and pTau fraction [F(1, 13) = 12.9, *P* = 0.00333] (**Figure 9B**). Iba1 fraction was significantly and positively correlated with pTau fraction [F(1, 13) = 10.2, *P* = 0.00696] (**Figure 9C**). Furthermore, the relationships between spatial reference memory in the PT in the Barnes maze test (number of visits to the goal region) and the above parameters were analyzed. The number of visits to the goal region was significantly and negatively correlated with Aβ42 fraction [F(1, 13) = 10.7, *P* = 0.00607] (**Figure 9D**), Iba1 fraction [F(1, 13) = 7.7, *P* = 0.01563] (**Figure 9E**), and pTau fraction [F(1, 13) = 11.9, *P* = 0.00431] (**Figure 9F**).

## Discussion

### Pathology in the Mouse AD Model

In the current study, we used a new mouse model of AD, the *App^NL-G-F^* mice that carries three *App* knock-in mutations associated with familial AD. This knock-in approach allows to express APP at a similar level to WT mice, and to generate elevated levels of pathogenic Aβ (Aβ42). Thus, it is unlikely that the potential artifacts due to APP overexpression occur in this mouse model (Saito et al., 2014). In this mouse model, (1) cortical Aβ deposition in the mice began by 2 months and was almost saturated by 7 months old, (2) the microgliosis and astrocytosis were observed at 9 months old, and (3) the memory impairment occurred by 6 months old (Saito et al., 2014). In the present study, astaxanthin supplementation to the experimental mice started from 5-to-6 weeks old before Aβ deposition started, and the mice were sacrificed at 9 months old so that the protective effects of astaxanthin on the onset and progression of AD could be analyzed.

In this model, we observed mild memory decline, accumulation of Aβ42 in the hippocampus and PFC, a mild increase in pTau fraction, and microglial accumulation (an increase in Iba1 fraction) in the *App^NL-G-F^* mice, which is consistent with the previous studies (Saito et al., 2014; Masuda et al., 2016; Hashimoto et al., 2019). These deficits in memory functions may not be ascribed to confounding effects outside the brain such as deficits in visual acuity and locomotor activity in the *App^NL-G-F^* mice since a previous study reported that motor and visual capabilities of *App^NL-G-F^* and WT mice were comparable at 24 months old (Sakakibara et al., 2019). We further indicated that 4-HNE protein adduct levels (a marker of lipid peroxidation), which were elevated in AD patients (Markesbery and Lovell, 1998; Zarkovic, 2003) and are toxic to normal cellular functions (Csala et al., 2015), were increased in the *App^NL-G-F^* mice. In addition, total GSH levels were decreased in the *App^NL-G-F^* mice, consistent with human AD patients (Mandal et al., 2015). A decrease in the PV-positive neuron density in the control-fed *App^NL-G-F^* mice may be ascribed to these changes in oxidative and anti-oxidant status due to Aβ42 accumulation in the control-fed *App^NL-G-F^* mice. Consistently, oligomers of Aβ42 have been reported to generate reactive oxygen species, which further induced membrane lipid peroxidation, intracellular Ca^2+^ entry associated with pore formation in the membrane, a decrease in membrane fluidity, and deficits in long-term potentiation (Yasumoto et al., 2019). The correlational analyses indicated that Aβ42 fraction was positively correlated with pTau and Iba1 fraction, while memory functions (number of visits to the goal region) were negatively correlated with Aβ and pTau fractions. These findings in this mouse AD model represent characteristics of human AD pathological findings and support the amyloid cascade theory of AD, in which accumulation of pathogenic Aβ induces amyloid plaques, hyperphosphorylation of tau (tauopathy), and microglial activation (Hardy and Selkoe, 2002; Selkoe and Hardy, 2016; Sasaguri et al., 2017; Edwards, 2019; Hashimoto et al., 2019).

### Protective Mechanisms of Astaxanthin

The present results indicated that astaxanthin decreased Aβ42 deposition and prevented memory decline in the *App^NL-G-F^* mice. Consistently, two recent studies reported that astaxanthin reduced Aβ40 levels in a 3xTg AD mouse model (Fanaee-Danesh et al., 2019) and reduced Aβ42 levels in rats with intracerebroventricular injections of Aβ42 (Rahman et al., 2019). The present study further indicated that astaxanthin decreased pTau and the Iba1 fraction, while it increased hippocampal PV-positive neuron density and total GSH levels. Furthermore, the correlation analyses showed that Aβ42 and pTau fractions were significantly negatively correlated with hippocampus-dependent cognitive functions. On the other hand, it is reported that astaxanthin crosses the blood-brain barrier (Grimmig et al., 2017) and is detectable in brain tissues after oral administration (Choi et al., 2011). These results provide clues to discuss several mechanisms in which astaxanthin suppressed the progression of AD in the *App^NL-G-F^* mice.

First, astaxanthin increased the hippocampal PV-positive neuron density in the astaxanthin-fed *App^NL-G-F^* mice, which may be attributed to an increase in total GSH in the astaxanthin-fed *App^NL-G-F^* mice. Previous studies reported that astaxanthin increased GSH biosynthesis through the nuclear factor erythroid-related factor 2 and the antioxidant responsive element (Nrf2-ARE) pathway in the rat brain with subarachnoid hemorrhage (Wu Q. et al., 2014), and also increased brain GSH levels in other brain disorders due to chemical oxidative stress and amygdalar kindling in rozdents (Wu W. et al., 2014; Lu et al., 2015). GSH is an endogenous antioxidant that protects body tissues from oxidative damages, while PV-positive neurons were sensitive to oxidative stress (see Introduction). Therefore, elevated levels of GSH may increase the PV-positive neuron density in the astaxanthin-fed *App^NL-G-F^* mice. Second, PV-positive neurons play a critical role in the generation of gamma oscillations (Bartos et al., 2007; Sohal et al., 2009; Nguyen et al., 2011; Nakamura et al., 2015). In the AD mouse model, as well as AD patients, reduction of gamma oscillations and dysfunctions of PV-positive neurons were reported (Stam et al., 2002; Verret et al., 2012). A recent study reported that optogenetic or sensory induction of gamma oscillations resulted in reduction of Aβ peptides in the hippocampus of a mouse model of AD (5XFAD mice), which was attributed to microglial activation and resultant increase in microglial uptake of Aβ (Iaccarino et al., 2016). Thus, PV-positive neurons may reduce Aβ levels through its effect on microglia. In the present study, astaxanthin decreased Iba1 fraction in the *App^NL-G-F^* mice. Since microglia accumulate around Aβ deposition (Hellwig et al., 2015), a decrease in Iba1 fraction may be attributed to a decrease in Aβ42 deposition in the astaxanthin-fed *App^NL-G-F^* mice. On the other hand, the fraction ratios of Iba1 against Aβ42 were greater in the astaxanthin-fed *App^NL-G-F^* mice than the control-fed *App^NL-G-F^* mice. This suggests that microglia were more strongly activated and sensitive to Aβ deposition in the astaxanthin-fed *App^NL-G-F^* mice. This activation of microglia, which may be attributed to gamma oscillation by PV-positive neurons (see above), may decrease Aβ deposition in the astaxanthin-fed *App^NL-G-F^* mice. Third, astaxanthin decreased pTau fraction in the astaxanthin-fed *App^NL-G-F^* mice compared with the control-fed *App^NL-G-F^* mice. In the present study, the pTau fraction was positively correlated with Aβ42 fraction, which is consistent with the amyloid cascade theory. These findings suggest that astaxanthin decreased pTau levels through its effects on Aβ42. Furthermore, recent studies reported that astaxanthin promoted Nrf2/ARE signaling in various experimental models (Li et al., 2013; Wu Q. et al., 2014; Zhu et al., 2018), while Nrf2 signaling reduces pTau by activating autophagy-mediated degradation of pTau in the mouse brain (Jo et al., 2014). These findings suggest that astaxanthin may also reduce pTau through its effects on autophagy.

It has been recommended that anti-Aβ treatments should be tested in and applied to patients in an early phase of AD, before the formation of Aβ plaque (i.e., patients without brain damage) (Perrin et al., 2009; Sperling et al., 2011; McDade and Bateman, 2017). Since astaxanthin extracted from *Haematococcus pluvialis* was widely supplied for human consumption as a safe natural compound (Ambati et al., 2014), the present findings suggest that astaxanthin could be applied to such aged people without dementia or those with family risks of AD prior to the onset of AD symptoms. Further studies are required to elucidate mechanisms of astaxanthin effects on Aβ pathology, and translational research studies using human subjects are also required to test the usefulness of astaxanthin in the prevention of AD.

In the present study, the WT mice were fed only normal chow without 0.02% astaxanthin, but not normal chow with 0.02% astaxanthin. A previous study reported that feeding of 0.02% astaxanthin-containing diet for 8 weeks did not affect adult hippocampal neurogenesis in male WT C57BL/6J mice (Yook et al., 2016). Other studies also reported that administration of astaxanthin [80 mg/kg/day, oral gavage for 10 weeks (Yang et al., 2019); 25 mg/kg/day, oral gavage for 10 weeks (4 day/week) (Zhou et al., 2015)] did not affect spatial learning and memory in a Morris water maze test in WT mice. However, a higher dose of astaxanthin (0.5% astaxanthin-containing diet) for 8 weeks enhanced neurogenesis and improved special memory in male WT C57BL/6J mice (Yook et al., 2016). These findings suggest that feeding of normal chow with 0.02% astaxanthin might not affect spatial learning and memory in a Barnes maze test in WT mice although normal chow with astaxanthin in doses higher than 0.02% might enhance learning and memory even in WT mice. Further studies are required to investigate effects of astaxanthin on learning and memory functions in WT mice.

## Data Availability Statement

The datasets generated for this study are available on request to the corresponding author.

## Ethics Statement

The animal study was reviewed and approved by the Ethics Committee for Animal Experiments at the University of Toyama.

## Author Contributions

HisN and KT designed the experiment. NH and YT performed the experiment. NH, YT, and HisN analyzed the data and wrote the manuscript. NH, YT, HisN, HirN, JM, KT, TS, and TCS revised the manuscript. All authors discussed the results, and approved the final manuscript.

## Funding

This study was supported partly by research funds from Fuji chemical industries Co., Ltd., and University of Toyama. The funder had no role in study design, data collection and analysis, decision to submit the paper, or preparation of the manuscript.

## Conflict of Interest

This study was supported partly by research funds from Fuji chemical industries Co., Ltd. Astaxanthin was provided from Fuji chemical industries Co., Ltd. NH is an employee of Fuji chemical industries Co., Ltd.

The remaining authors declare that the research was conducted in the absence of any commercial or financial relationships that could be construed as a potential conflict of interest.
